# Preterm Intraventricular Hemorrhage-Induced Inflammatory Response in Human Choroid Plexus Epithelial Cells

**DOI:** 10.3390/ijms22168648

**Published:** 2021-08-11

**Authors:** Zsolt Fejes, Marianna Pócsi, Jun Takai, Judit Erdei, Andrea Tóth, Enikő Balogh, Ágnes Rusznyák, Ferenc Fenyvesi, Andrea Nagy, János Kappelmayer, Viktória Jeney, Béla Nagy

**Affiliations:** 1 Department of Laboratory Medicine, Faculty of Medicine, University of Debrecen, H-4032 Debrecen, Hungary; fejes.zsolt@med.unideb.hu (Z.F.); pocsi.marianna@med.unideb.hu (M.P.); jun_takai@hotmail.com (J.T.); kappelmayer@med.unideb.hu (J.K.); 2 MTA-DE Lendület Vascular Pathophysiology Research Group, Research Centre for Molecular Medicine, Faculty of Medicine, University of Debrecen, H-4032 Debrecen, Hungary; jutka.erdei@gmail.com (J.E.); andrea.toth@med.unideb.hu (A.T.); balogh.eniko@med.unideb.hu (E.B.); jeneyv@belklinika.com (V.J.); 3 Department of Pharmaceutical Technologies, Faculty of Pharmacy, University of Debrecen, H-4032 Debrecen, Hungary; rusznyak.agnes@pharm.unideb.hu (Á.R.); fenyvesi.ferenc@pharm.unideb.hu (F.F.); 4 Doctoral School of Pharmaceutical Sciences, University of Debrecen, H-4032 Debrecen, Hungary; 5 Department of Pediatrics, Faculty of Medicine, University of Debrecen, H-4032 Debrecen, Hungary; nagyand@med.unideb.hu

**Keywords:** cerebrospinal fluid, choroid plexus epithelial cell, heme, inflammation, intraventricular hemorrhage, microRNA, oxidized hemoglobin

## Abstract

Following an intraventricular hemorrhage (IVH), red blood cell lysis and hemoglobin (Hb) oxidation with the release of heme can cause sterile neuroinflammation. In this study, we measured Hb derivates and cellular adhesion molecules ICAM-1 and VCAM-1 with cell-free miRNAs in cerebrospinal fluid (CSF) samples obtained from Grade-III and Grade-IV preterm IVH infants (IVH-III and IVH-IV, respectively) at multiple time points between days 0–60 after the onset of IVH. Furthermore, human choroid plexus epithelial cells (HCPEpiCs) were incubated with IVH and non-IVH CSF (10 *v*/*v* %) for 24 h in vitro to investigate the IVH-induced inflammatory response that was investigated via: (i) HMOX1, IL8, VCAM1, and ICAM1 mRNAs as well as miR-155, miR-223, and miR-181b levels by RT-qPCR; (ii) nuclear translocation of the NF-κB p65 subunit by fluorescence microscopy; and (iii) reactive oxygen species (ROS) measurement. We found a time-dependent alteration of heme, IL-8, and adhesion molecules which revealed a prolonged elevation in IVH-IV vs. IVH-III with higher miR-155 and miR-181b expression at days 41–60. Exposure of HCPEpiCs to IVH CSF samples induced HMOX1, IL8, and ICAM1 mRNA levels along with increased ROS production via the NF-κB pathway activation but without cell death, as confirmed by the cell viability assay. Additionally, the enhanced intracellular miR-155 level was accompanied by lower miR-223 and miR-181b expression in HCPEpiCs after CSF treatment. Overall, choroid plexus epithelial cells exhibit an abnormal cell phenotype after interaction with pro-inflammatory CSF of IVH origin which may contribute to the development of later clinical complications in preterm IVH.

## 1. Introduction

An intraventricular hemorrhage (IVH) is a severe, frequently seen complication in premature infants that results in an increased risk of neonatal mortality and neurodevelopmental impairments [1]. IVH affects about 15–20% of preterm infants with a very low birth weight (<1500 g) and surviving infants often develop post-hemorrhagic ventricular dilatation and other disabilities such as visual and hearing impairments, epilepsy, or cerebral palsy [1]. In these patients, the capillary network is immature and fragile in the germinal matrix, and periventricular regions are particularly vulnerable to spontaneous bleeding due to the rupture of the vasculature [2]. The severity of IVH ranges from mild to severe forms, namely Grades I–IV; the classification specifies IVH as Grade III (IVH-III) when with the presence of ventricular dilation and as Grade IV (IVH-IV) when with the extension to a concomitant intraparenchymal hemorrhage [3].

Following IVH, extravasated blood accumulates in the intraventricular space, causing sterile inflammation with elevation in pro-inflammatory cytokines such as interleukin-8 (IL-8), tumor necrosis factor alpha (TNF-α), and IL-1β, and causing an enhanced expression of adhesion receptors such as intercellular adhesion molecule-1 (ICAM-1), vascular adhesion molecule-1 (VCAM-1), and E-selectin [4]. In the same manner, soluble forms of these adhesive molecules were observed at a high quantity in cerebrospinal fluid (CSF) samples from patients with a subarachnoid hemorrhage (SAH) [5] and those with bacterial sepsis and meningitis [6].

After the hemolysis of the accumulated blood, hemoglobin (Hb) is released into the subarachnoid space and the cell-free Hb in turn undergoes oxidation, forming various oxidized metabolites such as methemoglobin (metHb, Fe^3+^) and ferrylhemoglobin (ferrylHb, Fe^4+^) with a subsequent liberation of heme due to the Hb denaturation [7]. These oxidized Hb forms and free heme exert pro-oxidant and pro-inflammatory effects on various cell types, e.g., glia cells [8], choroid plexus epithelial cells [9], and brain microvascular endothelial cells (ECs) [10]. Heme induces EC activation with lipid peroxidation and programmed cell death via a Toll-like receptor 4 (TLR4)-mediated signaling [11], and causes the hypersecretion of CSF in choroid plexus epithelial cells after posthemorrhagic hydrocephalus [12], leading to the barrier dysfunction of choroid plexus and the ependymal lining of the ventricles [13].

MicroRNAs (miRNAs) are small non-coding RNAs that participate in the post-transcriptional regulation of gene expression through promoting messenger RNA (mRNA) degradation that leads to the interference and attenuation of target protein expressions [14]. Among numerous functions, miRNAs have been demonstrated to regulate the inflammatory response via the NF-κB pathway in ECs [15]. For instance, miR-223 regulates ICAM-1 expression [16] and elevated miR-155 induces the production of pro-inflammatory interleukins such as IL-8 [17], while miR-181b modulates VCAM-1 and E-selectin on the endothelium [18]. The role of miRNAs has also been studied in the central nervous system (CNS) and is implicated in the development of different neurological disorders [19]. Upon brain injury, miRNAs are liberated from damaged tissues and cerebral vasculature into the subarachnoid space causing altered levels of circulating miRNAs in the CSF [20]. Increased CSF miR-21 and miR-221 levels have been linked to delayed cerebral ischemia (DCI) in adults with a subarachnoid hemorrhage (SAH) [21], while reduced miR-152 showed anti-inflammatory properties after intracerebral hemorrhage [22]. Our group has recently investigated the expression of some pro-inflammatory miRNAs in CSF specimens of preterm infants after IVH as potential prognostic biomarkers [23].

In this study, our goal was to perform quantitative assays to characterize the oxidized Hb content and heme level with adhesion molecules and cell-free miRNAs in CSF samples obtained from preterm IVH-III and IVH-IV subjects in connection to induced inflammatory milieu. We further applied some of these ex vivo IVH CSF specimens to investigate the pro-oxidant and pro-inflammatory effects of IVH on human choroid plexus epithelial cells (HCPEpiC) in vitro. For this purpose, primary HCPEpiCs were treated with IVH and non-IVH control CSF specimens to analyze the expression of pro-inflammatory mediators, reactive oxygen species (ROS) production, and the activation of the NF-κB pathway. Finally, inflammation-dependent miRNAs were quantified within CSF-treated HCPEpiCs to compare with the extracellular miRNA levels measured in post-IVH CSF.

## 2. Results

### 2.1. Characterization of the CSF Content between IVH-III and IVH-IV

**Figure 1:**
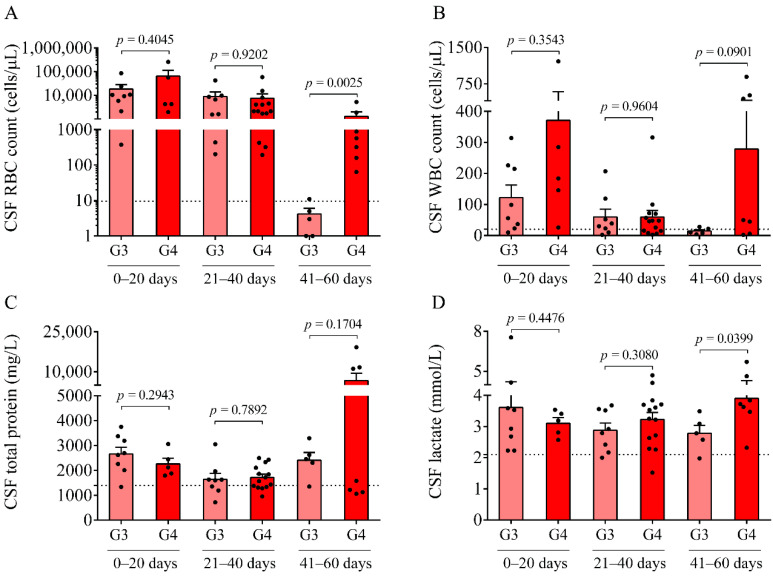
Comparison of routine laboratory parameters of Grade-III (G3, light red) and Grade-IV (G4, red) IVH groups at different time intervals after IVH onset. At 41–60 days, RBC count (**A**) and lactate concentration (**D**) were significantly elevated in the Grade-IV IVH group compared to the IVH-III group in the presence of a seemingly higher WBC count (**B**) and total protein (**C**) levels. Dots represent single values. Mean ± SEM are depicted. Dotted lines depict the mean value of the particular parameter measured in preterm non-IVH control samples [23]. To compare the data of the two groups, the Mann–Whitney U test or unpaired *t*-test was applied. Abbreviations: CSF, cerebrospinal fluid; G3, Grade-III IVH; G4, Grade-IV IVH; IVH, intraventricular hemorrhage; RBC, red blood cell; and WBC, white blood cell.

No significant difference was observed in the peripheral blood and serum parameters among the three study groups (see Table 1). As CSF samples were originally received for diagnostic purposes at multiple time points after the onset of the disease, IVH patients with Grade-III and Grade-IV were further divided into three subgroups based on the onset of symptoms (0–20 days, 21–40 days, and 41–60 days). Despite the different extent of disease severity, at early time points, i.e., at days 0–20 of post-IVH, no difference was observed in CSF RBC, the WBC counts, nor the total protein (Figure 1A–C) or S100B levels between IVH-III and IVH-IV groups. In contrast, there was a significantly higher RBC count (Figure 1A) and lactate level (Figure 1D) at days 41–60 in the IVH-IV cohort compared to IVH-III individuals (*p* = 0.0025 and *p* = 0.0399, respectively). Although the WBC count and total protein level (Figure 1B,C) were also higher in the CSF obtained from Grade-IV vs. Grade-III of IVH within the same intervals, the difference did not reach a statistical significance. These data indicate more severe long-term CSF abnormalities in Grade-IV IVH conditions than in Grade-III IVH.

### 2.2. Time-Dependent Alteration of Oxidized Hb Forms and Heme in Post-IVH CSF Samples

**Figure 2:**
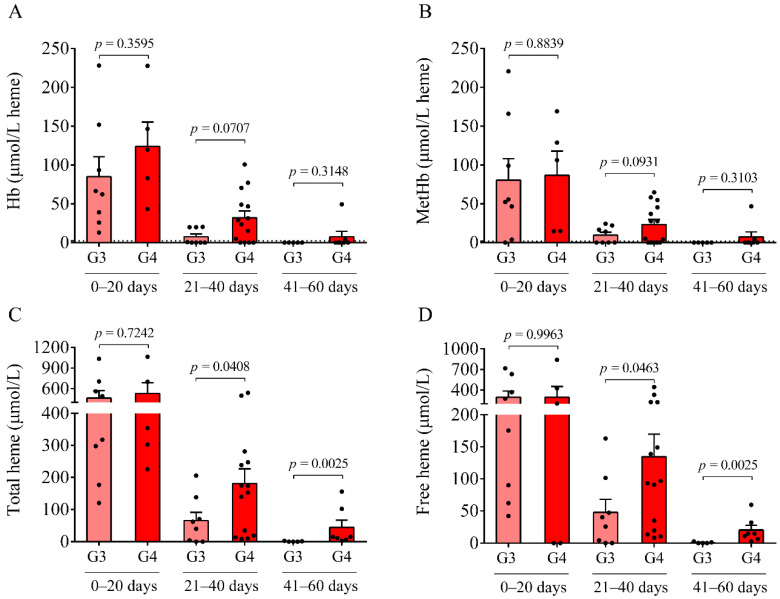
Analysis of the levels of Hb, metHb, and total and free heme in IVH-III (G3) and IVH-IV (G4) CSF samples at different time intervals after the disease onset. A time-dependent alteration of Hb (**A**), metHb (**B**), and total and free heme (**C**,**D**) was uncovered in post-IVH CSF samples, which was more pronounced in Grade-IV IVH patients (G4, red) compared to the Grade-III group (G3, light red). Dots represent single values. Mean ± SEM are depicted. Dotted lines depict the mean value of the particular parameter measured in preterm non-IVH control samples [23]. Mean total heme was as low as 0.8 μmol/L and free heme was practically undetectable in the control samples, and this is why the dotted lines cannot be recognized in parts C and D. To compare the data of the two groups, the Mann–Whitney U test or unpaired *t*-test was applied. Abbreviations: CSF, cerebrospinal fluid; G3, Grade-III IVH; G4, Grade-IV IVH; Hb, hemoglobin; IVH, intraventricular hemorrhage; and metHb, methemoglobin.

Besides routinely available laboratory parameters, the concentrations of Hb, metHb, ferrylHb, and heme were determined in IVH CSF specimens (Figure 2A–D). A time-dependent alteration of Hb, metHb, and heme (Figure 2A–D), as well as ferrylHb was demonstrated in both IVH groups. Importantly, a significantly larger amount of total (*p* = 0.0408 and *p* = 0.0463, respectively) and free heme (*p* = 0.0025 and *p* = 0.0025, respectively) was observed at 21–40 days of IVH in the IVH-IV vs. IVH-III cohort after 41–60 days of IVH (Figure 2C,D). These results underline that Grade-IV of IVH was associated with a higher degree of hemolysis and heme release into the CSF than Grade-III IVH.

### 2.3. Sustained Elevation in Soluble Pro-Inflammatory Protein Markers in the CSF after IVH-IV

**Figure 3:**
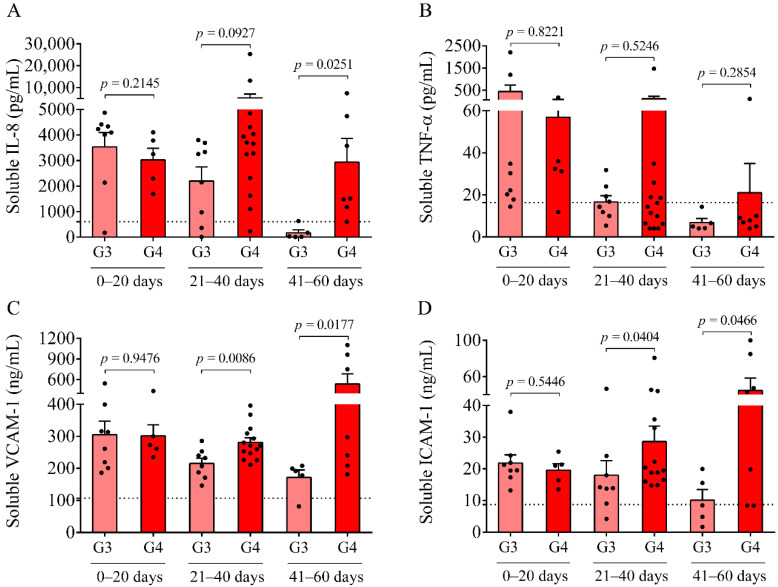
Measurement of soluble pro-inflammatory protein biomarkers in post-IVH CSF samples. Soluble IL-8 (**A**), TNF-α (**B**), VCAM-1 (**C**), and ICAM-1 (**D**) concentrations in IVH-III (G3, light red) were the highest in the CSF samples obtained between 0 and 20 days after the onset of IVH and showed a time-dependent decrease in the following intervals. The level of these markers remained elevated and even higher soluble IL-8 (**A**), VCAM-1 (**C**), and ICAM-1 (**D**) values were measured in IVH-IV (G4, red) CSF compared to the IVH-III at days 41–60. Dots represent single results. Mean ± SEM are depicted. Dotted lines depict the mean value of the particular parameter measured in preterm non-IVH control samples [23]. To compare the data of the two groups, the Mann–Whitney U test or unpaired *t*-test was applied. Abbreviations: CSF, cerebrospinal fluid; G3, Grade-III IVH; G4, Grade-IV IVH; ICAM-1, intercellular adhesion molecule 1; IL-8, interleukin-8; IVH, intraventricular hemorrhage; TNF-α, tumor necrosis factor alpha; and VCAM-1, vascular cell adhesion molecule 1.

In parallel to the analysis of oxidized Hb forms and heme, inflammation-dependent soluble biomarkers, i.e., IL-8, TNF-α, VCAM-1, and ICAM-1, were studied in post-IVH CSF samples. IL-8, VCAM-1, and ICAM-1 were significantly increased in both IVH cohorts and non-IVH controls, and TNF-α was also upregulated in the IVH cohorts in contrast to non-IVH controls (123.2 ± 60.1 vs. 16.3 ± 12.1 pg/mL, *p* < 0.0001). At days 0–20, no difference was found in the level of these parameters between IVH-III and IVH-IV (Figure 3A–D). Conversely, remarkably higher concentrations of VCAM-1 (*p =* 0.0086 and *p =* 0.0177, respectively) and ICAM-1 (*p* = 0.0404 and *p =* 0.0466, respectively) (Figure 3C,D) were determined in the IVH-IV subcohort compared to IVH-III subcohort following 21 days of IVH, while IL-8 was significantly elevated only at days 41–60 (*p* = 0.0251) (Figure 3A). In addition, we show a strong correlation between the total heme and soluble IL-8 (r = 0.7214, *p* < 0.0001), TNF-α (r = 0.7195, *p* < 0.0001), VCAM-1 (r = 0.5445, *p* < 0.0001), and ICAM-1 (r = 0.4650, *p* = 0.0001) concentrations in post-IVH CSF samples (Supplementary Figure S1).

### 2.4. Detection of Higher CSF miRNA Levels after IVH-IV Than IVH-III

**Figure 4:**
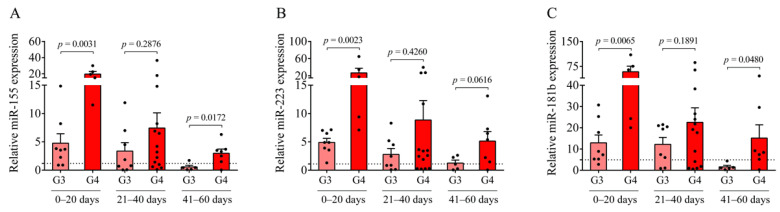
The expression of cell-free miRNAs between Grade-III (G3, light red) and Grade-IV (G4, red) preterm IVH groups. In CSF samples collected between 0 and 20, and 41 and 60 days after the onset of IVH, miR-155 (**A**), miR-223 (**B**), and miR-181b (**C**) were elevated in the grade-IV IVH group compared to the IVH-III group. Dots represent single expression values. Mean ± SEM are depicted. Dotted lines depict the mean value of the particular extracellular miRNA analyzed in preterm non-IVH control samples [23]. To compare the data of two groups, the Mann–Whitney U test or unpaired *t*-test was applied. Abbreviations: CSF, cerebrospinal fluid; G3, Grade-III IVH; G4, Grade-IV IVH; IVH, intraventricular hemorrhage; and miRNA, microRNA.

Next, we investigated the expression of pro-inflammatory miR-155, miR-223, and miR-181b in the respective IVH conditions, which are miRNA subtypes that play an essential role in the post-transcriptional gene regulation upon inflammation in ECs [15,16,17,18]. First, extracellular miRNAs in CSF samples from IVH individuals were analyzed to evaluate their potential as laboratory markers and to contrast the two IVH severity groups in terms of the miRNA profile (Figure 4A–C). In contrast to Hb products and soluble protein biomarkers (Figure 2 and Figure 3), circulating miR-155, miR-223, and miR-181b levels in the CSF were more induced in Grade-IV compared to Grade-III IVH patients regardless of the sampling time points. Moreover, significantly higher levels of miR-155 (*p* = 0.0172) and miR-181b (*p* = 0.0480) were analyzed in IVH-IV following 41–60 days after the onset compared to IVH-III (Figure 4A–C). Accordingly, it is indicative of the potential significance of cell-free CSF miR-155, miR-223, and miR-181b to differentiate the different severity of IVH as a potential laboratory parameter at specific time intervals of post-IVH.

### 2.5. Pro-Oxidant and Pro-Inflammatory Effects of Post-IVH CSF on Choroid Plexus Epithelial Cells

IVH is characterized by sterile inflammation with an elevated level of released cytokines, Hb oxidation products, and cellular adhesion molecules [2,10]. As these mediators have been characterized in the post-IVH CSF samples above, we next addressed the pro-oxidant and pro-inflammatory effects of the CSF with different IVH origins on choroid plexus epithelial cells. To achieve this purpose, HCPEpiCs were exposed to IVH-III and IVH-IV and to non-IVH control CSF samples (10 *v*/*v* %) for 24 h among in vitro conditions. CSF samples that were used for in vitro experiments showed a substantial heme content (mean ± SEM, 164.4 ± 76.5 μmol/L in Grade-III vs. 292.1 ± 127.7 μmol/L in Grade-IV) and high TNF-α level (17.6 ± 3.8 ng/mL in Grade-III vs. 20.4 ± 3.9 ng/mL in Grade-IV) in contrast to non-IVH control samples with undetectable heme and minimal TNF-α concentration (4.4 ± 0.4 ng/mL).

**Figure 5:**
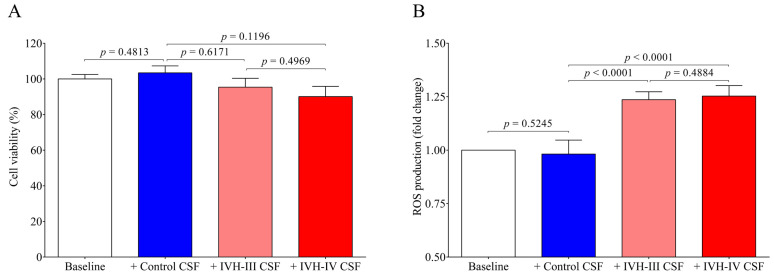
Cell viability test and the detection of ROS production induced by ex vivo CSF samples obtained from IVH-III (light red) patients, IVH-IV (red) patients, or non-IVH clinical controls (blue). In the cell viability assay (**A**), HCPEpiCs were treated with ex vivo CSF samples (10 *v*/*v* %) for 24 h, followed by an MTT-based cell viability test. Results were shown as a percentage of viability of vehicle-treated (baseline, clear column) cells. (B) Confluent HCPEpiCs were exposed to vehicle non-IVH CSF, IVH-III, or IVH-IV CSF samples (10 *v*/*v* %) for 4 h and ROS production was measured after every 30 min using the CM-H2DCFDA assay for 3 h. The graphs show the ROS production at the 60 min time point expressed in fold change (**B**). Mean ± SEM values are depicted, *n* = 7–8/group. The ANOVA test with Bonferroni’s multiple comparison test was used for the comparisons. Abbreviations: CSF, cerebrospinal fluid; CM-H2DCFDA, General Oxidative Stress Indicator assay; HCPEpiCs, human choroid plexus epithelial cells; IVH, intraventricular hemorrhage; MTT, Thiazolyl Blue Tetrazolium Bromide; and ROS, reactive oxygen species.

First, to investigate whether treatment with IVH-III and IVH-IV CSF samples at this volume ratio results in any cellular death, a cell viability test was performed with ex vivo CSF samples (10 *v*/*v* %) which confirmed that incubation with neither Grade-III nor Grade-IV IVH CSF affected cell viability at this cell culture medium volume (*p* = 0.6171 and *p* = 0.1196, respectively) (Figure 5A). Additionally, some experiments were performed with CSF treatment of up to 30 *v*/*v* %. We found that IVH-IV, but not IVH-III CSF, significantly affected cell viability compared to the untreated baseline samples (*p* = 0.0279) (data not shown). Based on these data, we can speculate that IVH-derived CSF consisting of even larger amounts of heme can result in not only the activation of choroid plexus epithelial cells but also cell death after the onset of preterm IVH. Gram et al. [9] earlier investigated cell viability of choroid plexus cell cultures after being exposed to 1 to 30% of hemorrhagic CSF for 4–24 h and after a 4 h-treatment a moderate, dose-dependent cell death was observed, although almost complete cell death was described based on LDH release at 24 h [9].

As the role of heme has been implicated in the mechanism of ROS generation under hemolytic conditions [7], the further question of whether IVH-derived CSF samples could induce ROS production in HCPEpiCs was investigated. HCPEpiCs were treated with IVH-III and IVH-IV CSF samples for 1 h and 4 h, and the production was already observed after the first 30-min measurement. In addition, significantly higher ROS generation (*p* < 0.0001) was detected at 60 min (Figure 5B). These findings confirmed that, methodologically, cell death was not provoked by this CSF treatment and suggested that ROS production was induced by the high heme content of the CSF.

**Figure 6:**
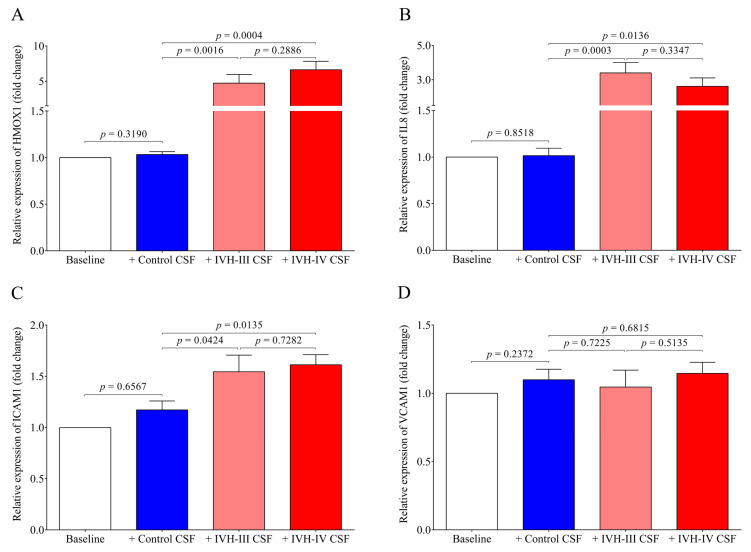
Induction of HMOX1 (**A**), IL8 (**B**), and ICAM1 (**C**) mRNA expression in HCPEpiCs treated with the non-IVH control CSF (blue column) compared to HCPEpiCs exposed to IVH-III (light red) or IVH-IV (red) CSF samples (10 *v*/*v* %). HMOX1, IL8, and ICAM1 mRNA expression revealed profound upregulation after exposure to IVH-III and IVH-IV CSF (A–C); however, interestingly, no change was observed in the VCAM1 mRNA level (**D**) after 24 h. Mean ± SEM values are depicted, *n* = 7–8/group. The ANOVA test with Bonferroni’s multiple comparison test was used for the comparisons. Abbreviations: CSF, cerebrospinal fluid; HCPEpiCs, human choroid plexus epithelial cells; HMOX1, heme oxygenase 1; ICAM1, intercellular adhesion molecule 1; IL8, interleukin 8; IVH, intraventricular hemorrhage; mRNA, messenger RNA; and VCAM1: vascular cell adhesion molecule 1.

As the possibility of the detrimental effect of the CSF treatment on cell viability was excluded, pro-oxidant and pro-inflammatory gene expression was further quantified in vitro under the same treatment conditions. Heme oxygenase 1 (gene name *HMOX1*), the stress-responsive inducible enzyme that catalyzes heme degradation upon heme overload conditions [7], was significantly induced by both IVH-III (*p* = 0.0016) and IVH-IV CSF samples (*p* = 0.0004), while non-IVH CSF showed no change as compared to untreated (baseline) cells (Figure 6A). In addition, IL8 (*p* = 0.0003 and *p* = 0.0136, respectively) (Figure 6B) and ICAM1 (*p* = 0.0424 and *p* = 0.0135, respectively) (Figure 6C) mRNAs were augmented by IVH-III and IVH-IV CSF vs non-IVH control CSF, while surprisingly, VCAM1 mRNA did not show any alteration after treatment with IVH CSF (Figure 6D). There was no difference in the expression of all the four mRNAs between Grade-III or Grade-IV CSF treatments (Figure 6A–D). These data imply that cellular activation is triggered in choroid plexus epithelial cells via interaction with the CSF containing oxidative Hb products and different pro-inflammatory mediators following IVH.

### 2.6. Activation of the Pro-Inflammatory NF-κB Pathway in HCPEpiCs by IVH CSF In Vitro

**Figure 7:**
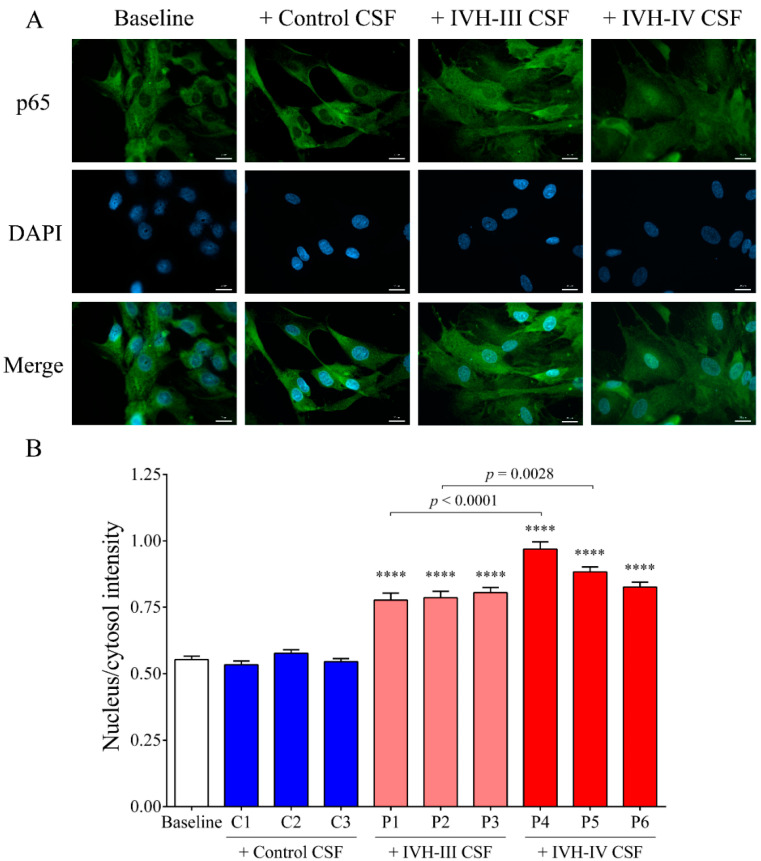
NF-κB pathway activation via p65 nuclear translocation was visualized by immunofluorescence staining in HCPEpiCs (**A**) and the ratio of the fluorescence intensity of the NF-κB immunostaining in cell nuclei and cytosol was analyzed (**B**). Representative images of vehicle (baseline) or ex vivo CSF-treated cells are shown (**A**). Specific p65 staining: green; cell nuclei: blue (DAPI). Scale bar: 20 µm (**A**). When HCPEpiCs were exposed to IVH-III (light red, P1–P3) or IVH-IV (red, P4–P6) CSF for 1 h, the nucleus/cytosol intensity of the p65 staining was significantly increased compared to non-IVH controls (blue, C1–C3) (**B**). Mean ± SEM values are depicted, *n* = 15–25 cells were analyzed/group. **** *p* < 0.0001 vs. control samples based on the ANOVA test with Bonferroni’s multiple comparison test. Abbreviations: CSF, cerebrospinal fluid; HCPEpiCs, human choroid plexus epithelial cells; IVH, intraventricular hemorrhage; and NF-κB, nuclear factor-kappa B.

To establish the relationship between NF-κB signaling activation due to IVH-induced pro-inflammatory conditions in choroid plexus epithelial cells, the activity of the NF-κB pathway was monitored by p65 nuclear translocation assessment using fluorescent microscopy. The p65, also known as RELA, is one of the five components that constitute the NF-κB transcription factor family and is used as a marker of the activated NF-κB pathway. IVH CSF-treated HCPEpiCs were incubated with three randomly selected non-IVH (C1–C3), IVH-III (P1–P3), and IVH-IV (P4–P6) CSF specimens (10 *v*/*v*%) for 1 h. Nuclear translocation of p65 was visualized by immunofluorescence staining in HCPEpiCs, followed by an analysis of the ratio of the fluorescence intensity of the NF-κB immunostaining in cell nuclei and cytosol (Figure 7A,B). No intranuclear staining of p65 was observed after the non-IVH CSF treatment as compared to baseline conditions (Figure 7A). As expected, IVH-III and IVH-IV CSF induced the internalization of the p65 subunit into the nuclei (*p* < 0.0001) (Figure 7B) and even greater p65 positivity was quantified in two randomly selected sets of IVH-IV CSF samples (P1 vs. P4, *p* < 0.0001 and P2 vs. P5, *p* = 0.0028) compared to the IVH-III CSF counterparts (Figure 7B).

**Figure 8:**
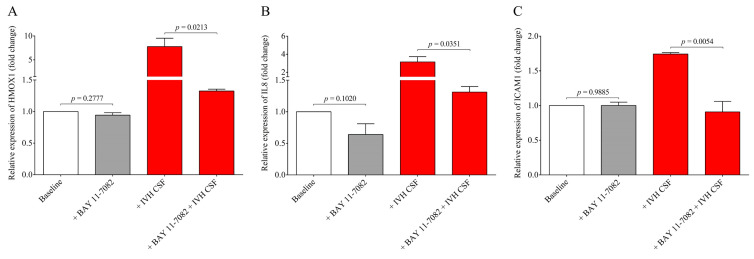
Investigation of the NF-κB pathway-dependent inflammatory response in HCPEpiCs using a specific inhibitor. HCPEpiC cells were pretreated with the NF-κB pathway inhibitor BAY 11-7082 (5 µM) for 4 h (gray bar) and then exposed to IVH CSF (red bar) samples (10 *v*/*v* %) for 24 h. In the presence of BAY 11-7082, we detected significantly lower HMOX1 (**A**), IL8 (**B**), and ICAM1 (**C**) mRNA levels than samples with IVH CSF alone. The inhibitor alone did not alter these levels compared to the baseline samples. Mean ± SEM values are depicted, *n* = 3–4/group. The ANOVA test with Bonferroni’s multiple comparison test was used for comparison. Abbreviations: CSF, cerebrospinal fluid; HCPEpiCs, human choroid plexus epithelial cells; HMOX1, heme oxygenase 1; ICAM1, intercellular adhesion molecule 1; IL8, interleukin 8; IVH, intraventricular hemorrhage; mRNA, messenger RNA; and NF-κB, nuclear factor-kappa B.

To provide further evidence on the involvement of the NF-κB pathway in IVH-mediated cellular activation, a specific NF-κB pathway inhibitor, BAY 11-7082, was used to prevent the activation of this signaling in HCPEpiCs under IVH-specific conditions. For this purpose, cell cultures were preincubated with BAY 11-7082 for 4 h and both non-IVH and IVH CSF specimens (10 *v*/*v* %) were added for 24 h (Figure 8). HMOX1, IL8, and ICAM1 mRNAs were quantified by RT-qPCR as described below. Treatment with BAY 11-7082 alone did not alter gene expression in a significant manner, while the NF-κB pathway inhibitor resulted in a significant decrease in HMOX1 (*p* = 0.0213), IL8 (*p* = 0.0351), and ICAM1 (*p* = 0.0054) mRNA levels that were induced by IVH CSF (Figure 8A–C). These results suggest that the pro-inflammatory components accumulated in the CSF after the onset of IVH promoted a remarkable inflammatory response and cellular activation in choroid plexus epithelial cells via the NF-κB pathway.

### 2.7. Alteration in miR-155, miR-223, and miR-181b Levels in HCPEpiCs after Treatment with IVH CSF

**Figure 9:**
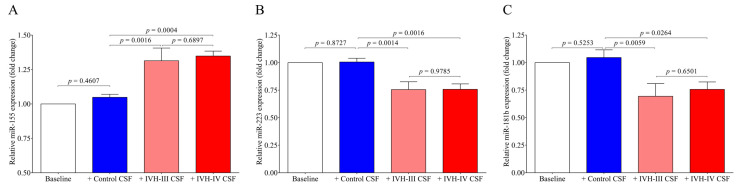
Intracellular expression of inflammation-specific miR-155 (**A**) was upregulated in response to the exposure of HCPEpiCs to IVH-III (light red column) or IVH-IV (red column) CSF samples for 24 h. Conversely, miR-223 (**B**) and miR-181b (**C**) levels were downregulated by the inflammatory stimulus compared to cells treated with non-IVH CSF samples (blue column). Mean ± SEM are depicted, *n* = 7–8/group. The ANOVA test with Bonferroni’s multiple comparison test was used for the comparisons. Abbreviations: CSF, cerebrospinal fluid; HCPEpiCs, human choroid plexus epithelial cells; IVH, intraventricular hemorrhage; and miRNA, microRNA.

Finally, we quantified the intracellular levels of miR-155, miR-223, and miR-181b in HCPEpiCs after the treatment with IVH-III or IVH-IV CSF. The expression of inflammation-specific miR-155 was upregulated in response to IVH-III (*p* = 0.0016) or IVH-IV CSF (*p* = 0.0004) exposure by 24 h vs non-IVH control CSF (Figure 9A). In contrast, intracellular miR-223 (*p* = 0.0014 and *p* = 0.0016) and miR-181b levels (*p* = 0.0059 and *p* = 0.0264) were downregulated by the experimental stimulus vs the cells with non-IVH control CSF treatment (Figure 9B,C). Consequently, based on the formerly approved relationship between miR-223/ICAM1 [16], reduced miR-223 can contribute to up-regulated *ICAM1* expression detected at high protein levels in IVH CSF specimens (Figure 3D) and CSF-treated HCPEpiCs at the mRNA level (Figure 6C). Based on these findings, IVH-derived mediators in the CSF trigger an activation of epithelial cells into the inflammatory phenotype accompanied by a complex transcriptional and post-transcriptional machinery involving miRNA alterations.

## 3. Discussion

It has been known for a long time that inflammation has a critical role in the patho-mechanism of IVH-induced brain injury that may lead to irreversible neurodevelopmental complications, especially in those born before the 32 weeks of gestation [3,24]. However, the molecular mechanism regarding how IVH induces the inflammatory response and the dysfunction of choroid plexus in this disease has not been entirely clarified. It has been demonstrated that extravasation of RBCs into the intraventricular compartment and the subsequent oxidation of liberated Hb triggers a cascade of cellular events including the secretion of vasoactive and pro-inflammatory mediators [2,4,25]. Despite recent increasing interest, choroid plexus is still a relatively understudied tissue in CNS, although it is the main source of CSF production and is part of an important barrier against pro-inflammatory stimuli [26]. Therefore, further knowledge on choroid plexus and CSF alteration is required for better understanding the development and complications of preterm IVH.

In this study, we analyzed oxidized Hb content and heme level for the characterization of human CSF samples obtained in different intervals from preterm IVH-III and IVH-IV patients. To model IVH-induced cellular activation in choroid plexus following IVH, ex vivo CSF samples were added to HCPEpiCs to investigate pro-oxidant and pro-inflammatory impacts of the disease in vitro via the expression of pro-inflammatory mediators, ROS production, and the activation of the NF-κB pathway. In addition, inflammation-dependent intracellular miRNA levels were quantified in CSF-treated HCPEpiCs along with the extracellular miRNA expression measured in post-IVH CSF samples.

Our study has three major findings: (i) there were significantly higher levels of heme, IL-8, ICAM-1, and VCAM-1 in the CSF of IVH-IV subjects after 41–60 days of the onset of the disease with the presence of a more increased RBC count and increased lactate concentration compared to the CSF from IVH-III; (ii) treatment of HCPEpiCs with IHV-III or IVH-IV CSF triggered the inflammation-dependent expression of *IL8* and *ICAM1* with *HMOX1* genes via induced ROS production and NF-κB pathway activation; and (iii) cell-free CSF miR-155, miR-223, and miR-181b showed a much higher level in IVH-IV compared to IVH-III patients not only at days 41–60 but also in the early phase of the disease (days 0–20), while the intracellular form of these miRNAs as post-transcriptional regulators of the gene expression was also altered in HCPEpiCs as part of the inflammatory response.

Here, we studied the CSF of all 47 IVH (21 IVH-III and 26 IVH-IV) and 14 non-IVH individuals. As CSF samples were obtained for diagnostic purposes at various time points after the onset of the disease, IVH patients were further divided into three subgroups (0–20 days, 21–40 days, and 41–60 days). A significantly higher RBC count and lactate level were measured between days 41 and 60 in the IVH-IV cohort compared to IVH-III individuals. In addition, the WBC count and total protein level were higher in the CSF obtained from IVH-IV compared to IVH-III within the same intervals. These data indicate more severe long-term CSF abnormalities in Grade-IV IVH conditions. Importantly, a time-dependent alteration of Hb, metHb, and heme, as well as ferrylHb, was demonstrated in both IVH severity groups, in agreement with our former data in IVH-III subjects [10]. As a significantly larger amount of total and free heme was observed in the IVH-IV cohort compared to the IVH-III cohort after 41–60 days of IVH, it can be concluded that IVH-IV was associated with a higher degree of hemolysis and heme release into the CSF than the IVH-III.

In parallel, inflammation-dependent soluble biomarkers were studied in post-IVH CSF samples. Compared to our previous measurements in non-IVH CSF specimens [23], IL-8, VCAM-1, and ICAM-1 were significantly increased in both IVH cohorts and TNF-α was also upregulated compared to current non-IVH controls (123.2 ± 60.1 vs. 16.3 ± 12.1 pg/mL, *p* < 0.0001). At days 0–20, no difference was found in the level of these parameters between IVH-III and IVH-IV. In contrast, higher concentrations of VCAM-1 and ICAM-1 were determined in the IVH-IV compared to in the IVH-III subcohort following 21 days of IVH, while IL-8 was significantly elevated only at days 41–60. Moreover, a strong correlation between total heme and soluble IL-8, TNF-α, VCAM-1, and ICAM-1 concentrations was found in post-IVH CSF samples. Similarly, a significant positive correlation between total heme and VCAM-1, ICAM-1, and IL-8 has been reported in the CSF of IVH-III premature infants [10]. We have to add that due to the lack of ultrasound information on the exact volume of intraventricular blood after the onset of IVH, we could only correlate the level of these biomarkers with the grade of the disease. Interestingly, the CSF RBC and WBC count, concentration of heme, and oxidized Hb products, as well as IL-8 and TNF-α levels, were found within the ‘normal range’ in IVH-III CSF samples 41–60 days after the onset IVH (Figure 1A,B, Figure 2A–D and Figure 3A,B) based on the former data of preterm non-IVH control CSF samples [23].

Next, we investigated the expression of pro-inflammatory miR-155, miR-223, and miR-181b under the IVH condition, which plays an essential role in the post-transcriptional gene regulation upon inflammation in ECs [15,16,17,18]. In contrast to Hb products and soluble protein biomarkers, circulating miR-155, miR-223, and miR-181b levels in the CSF were more elevated in Grade-IV vs. Grade-III IVH patients regardless of the sampling time points. Moreover, significantly higher levels of miR-155 and miR-181b was analyzed in IVH-IV following 41–60 days of IVH compared to IVH-III. Accordingly, cell-free CSF miRNAs can be of substantial aid in differentiating the different severity of IVH. Increased miR-21 and miR-221 CSF levels have been linked to delayed cerebral ischemia (DCI) in adults with SAH [21], while reduced miR-152 has shown anti-inflammatory properties after an intracerebral hemorrhage [22]. Our group has recently investigated the expression of some pro-inflammatory miRNAs in CSF specimens of preterm infants after IVH as novel prognostic biomarkers [23].

IVH has been characterized by sterile inflammation with an elevated level of cytokines, oxidized and cross-linked Hb, free heme, and cellular adhesion molecules [2,10,25,27]. In addition, the presence of heme is a well-established danger-associated molecular pattern, which binds to TLR4 to induce immune responses [28]. In ECs, ferryl hemoglobin induces intercellular gap formation and decreases junctional resistance with enhanced monocyte adhesion [27], while heme can result in endoplasmic reticulum stress that is potentially involved in the hemolysis and hemorrhage-associated pathologies [29]. In the present study, we addressed the pro-oxidant and pro-inflammatory effects of the CSF with different IVH origins on choroid plexus epithelial cells. Hence, HCPEpiCs were exposed to IVH-III and IVH-IV compared to the non-IVH control CSF samples (10 *v*/*v* %) for 24 h among in vitro conditions. Cell viability was unaffected after incubation with IVH-III and IVH-IV CSF. Heme is a catalyst of the Fenton reaction and has been implicated in ROS generation under hemolytic conditions [7]. Substantial production of ROS in HCPEpiCs was detected in response to both types of the CSF sample for 4 h. Thereafter, pro-oxidant and pro-inflammatory gene expression was quantified under the same conditions. Expression of HMOX1, the stress-responsive inducible enzyme that catalyzes heme degradation upon heme overload [7], was significantly induced by either IVH-III or IVH-IV CSF samples, while non-IVH samples showed no change as compared to untreated cells. Exposure of glia cells with hemorrhagic CSF samples resulted in an increased rate in ROS production that positively correlated with the expression of pro-inflammatory chemokines [8]. Others recently investigated ROS changes in the CSF after lumbar spinal stenosis, which provided an early detection of this clinical status [30].

In our study, IL8 and ICAM1 mRNAs were also augmented, while, surprisingly, VCAM1 mRNA did not show any alteration in HCPEpiCs after the treatment with IVH CSF, in contrast to the elevated CSF VCAM-1 level in IVH. These data imply that apart from the triggered cellular activation of choroid plexus epithelial cells by the CSF containing oxidative Hb products and different cytokines, stimulation of other cell types such as intracerebral capillary endothelial cells may contribute to abnormal parameters of the CSF [10]. In addition, potential contamination of the CSF with peripheral blood containing a higher concentration of adhesion molecules may also explain this discrepancy. In concordance with our current data, former in vitro studies demonstrated the pro-inflammatory effects of Hb derivates (e.g., metHb) on primary rabbit astrocyte cell cultures [31] and choroid plexus epithelial cells [9], causing an elevated expression of TNF-α in the presence of an induced HMOX1 mRNA level [31] and caspase activation with structural disintegration of the cells at high agonist concentrations [9]. Moreover, released pro-inflammatory cytokines and adhesion molecules help to retain the leukocytes on the surface of choroid plexus epithelial cells after their exit from the vasculature. To date, several mechanisms have been reported to facilitate the transmigration of leukocytes across epithelial and endothelial cells into the ventricles after IVH [32].

We established the relationship between NF-κB signaling activation due to IVH-related pro-inflammatory conditions in choroid plexus epithelial cells via monitoring the activity of the NF-κB pathway with the p65 nuclear translocation by fluorescent microscopy. IVH CSF-treated HCPEpiCs were incubated with three randomly selected non-IVH, IVH-III, and IVH-IV CSF specimens (10 *v*/*v* %) for 1 h. No nuclear staining of p65 was seen after the non-IVH CSF treatment compared to the baseline conditions. As we expected, IVH-III and IVH-IV CSF samples induced the internalization of the p65 subunit into the nuclei and even greater p65 positivity was quantified in the case of two IVH-IV CSF samples compared to their IVH-III CSF counterparts. Former in vivo data supports our findings that TLR4/NF-κB signaling is activated under IVH in rat models of IVH [12,13]. To provide further evidence on the involvement of the NF-κB pathway in IVH-mediated cellular activation, a specific NF-κB pathway inhibitor, BAY 11-7082, was used to prevent the activation of this signaling in HCPEpiCs under IVH-specific conditions. Inhibition of the NF-κB pathway resulted in a significant decrease in HMOX1, IL8, and ICAM1 mRNA levels induced by IVH CSF. These results suggest that pro-inflammatory components accumulated in the CSF after the onset of IVH promoted a remarkable inflammatory response and cellular activation in choroid plexus epithelial cells via the NF-κB pathway.

Finally, we quantified the intracellular miRNAs in HCPEpiCs after the treatment with the CSF analyzed above. The expression of inflammation-specific miR-155 was upregulated in response to IVH-III or IVH-IV CSF treatment by 24 h, whereas intracellular miR-223 and miR-181b levels were downregulated by this stimulus in comparison to cells with non-IVH control CSF treatment. Consequently, it is explained by the formerly approved relationship between miR-223/ICAM1 [16] that reduced miR-223 can mediate up-regulated ICAM1 expression detected at high protein levels in IVH CSF specimens and CSF-treated HCPEpiCs at the mRNA level. Based on these findings, IVH-derived mediators in the CSF trigger an inflammatory phenotype of epithelial cells accompanied by a complex transcriptional and post-transcriptional machinery. Important to note, an opposite tendency was found in the level of intra and extracellular miR-223 and miR-181b based on the data of IVH CSF-treated HCPEpiCs and ex vivo CSF samples with IVH origins, respectively (Figure 4 and Figure 9). Although we did not investigate the background of this discrepancy, we formerly reported [23] that heme-treated HCPEpiCs showed an elevated expression of miR-223 and miR-181b in the supernatant; thus, an active release of these pro-inflammatory miRNAs may contribute to their lower intracellular level [23]. Recently, miR-367 was described to regulate IRAK4 in primary microglia cells that negatively modulated the inflammatory response of microglia in an intracerebral hemorrhage [33]. Based on an animal model of an intracerebral hemorrhage, the levels of miR-155 were amplified in different parts of the CNS and this alteration was accompanied by increases of IL-1β, IL-6, and TNF-α, thus blocking the central miR-155 pathway, which may play a beneficial role in regulating neurological function [34]. Taken together, further studies are required to reveal additional cellular mechanisms in the development of IVH-mediated inflammation.

## 4. Materials and Methods

### 4.1. Study Participants, Cerebrospinal Fluid Collection, and Routine Laboratory Analyses

In this study, 47 preterm infants (22 females and 25 males) were involved after a diagnosis with IVH-III (*n* = 21) or IVH-IV (*n* = 26) with a mean gestational age at birth of 29.1 ± 4.2 weeks and 27.5 ± 1.9 weeks, respectively (Table 1). As controls, we recruited 14 non-IVH preterm infants (8 females and 6 males) with a mean gestational age of 32.0 ± 6.8 weeks (Table 1) at delivery who were diagnosed with congenital hydrocephalus without bleeding or had a sterile CSF sample after being investigated via lumbar puncture testing CNS infection. Cranial ultrasound examination was performed at the time of laboratory analyses in all subjects to reveal the presence or absence of IVH. Grading of the disease was determined by the clinicians (Department of Pediatrics, University of Debrecen, Debrecen, Hungary). No statistical differences were found in the baseline demographical parameters such as age, sex, and birth weight between recruited non-IVH and IVH patients (Table 1).

CSF samples were collected by spinal tap or ventricular reservoir puncture at 28.9 ± 13.8 days after the onset of IVH. In parallel, 14 CSF specimens were obtained from non-IVH clinical controls. In this study, we examined the leftover of CSF specimens (*n* = 61) obtained for diagnostic purposes and no CSF was collected exclusively for inclusion. This study was approved by the Scientific and Research Ethics Committee of the University of Debrecen (permit number: 4876-2017, 25 September 2017) in accordance with the Declaration of Helsinki. Parental consent forms were signed by the parents of the infants involved in this study.

Within 30 min after collection, CSF samples were centrifuged at 650 g for 5 min at 4 °C and cell-free supernatants were immediately frozen at −80 °C until the analysis. Routine laboratory parameters in the CSF in addition to peripheral blood and serum samples were measured in the Department of Laboratory Medicine, University of Debrecen, Debrecen, Hungary). In CSF samples, RBC and WBC counts were analyzed on a Sysmex XN-1000 hematology analyzer (Sysmex, Kobe, Japan), while the CSF total protein level was determined by immunoturbidimetry and CSF lactate concentration was analyzed by a colorimetric test on a Cobas 6000 analyzer (Roche Diagnostics, Mannheim, Germany). The S100 calcium-binding protein B (S100B) level in the CSF was measured by a chemiluminescence immunoassay (Liaison XL, DiaSorin, Saluggia, Italy). Whole blood RBC, WBC, and platelet count, as well as Hb concentration, were determined by an Advia 2120 Hematology System analyzer (Bayer Diagnostics, Tarrytown, NJ, USA). Serum C-reactive protein (CRP) and procalcitonin (PCT) levels were measured by an electro-chemiluminescent immunoassay using a Cobas 8000 analyzer (Roche Diagnostics).

### 4.2. Determination of Hemoglobin Forms and Heme Levels in the CSF

The absorbance spectra (250–700 nm) of the CSF samples were measured using the spectrophotometer (NanoDrop, Thermo Scientific, Wilmington, DE, USA) as we have done recently [10,23]. Briefly, concentrations of Hb, metHb, and ferrylHb were calculated based on the absorbance values measured at 541, 576, and 630 nm using the absorption coefficients and equations determined by others previously [35]. Total heme concentration in the CSF samples was determined using a QuantiChrom Heme Assay Kit (Gentaur Ltd., London, UK) according to the manufacturer’s instructions. Concentration of free heme (non-Hb bound heme) was calculated by the following equation: free heme = total heme − Hb bound heme − metHb bound heme − ferrylHb bound heme.

### 4.3. Treatment of HCPEpiC Cells with the IVH CSF Samples

Primary human choroid plexus epithelial cells (HCPEpiC, ScienCell Research Laboratories, Carlsbad, CA, USA) were cultured in a special Epithelial Cell Medium (ScienCell) containing 2% fetal bovine serum (FBS, ScienCell), 1% Epithelial Cell Growth Supplement (ScienCell), and 1% Penicillin/Streptomycin solution (ScienCell) in a 5% CO_2_ humidified atmosphere (95%) at 37 °C. For culturing these cells, we followed the manufacturer’s protocol. Cell density was set to 5000 cells per cm^2^ and poly-L-lysine (ScienCell) coated (2 µg/cm^2^) BioLite cell culture flasks (Thermo Scientific, Rochester, NY, USA) were used. HCPEpiC cells (2 × 10^5^/well) at passages 4–6 were subcultured into BioLite 6-well plates (Thermo Scientific) and then treated with ex vivo IVH-III, IVH-IV, or non-IVH control CSF samples (10 *v*/*v* %) for 24 h. After treatment, cells were washed with Dulbecco’s Phosphate Buffered Saline (DPBS) solution (Lonza, Walkersville, MD, USA), then lysed in 1 mL TRI Reagent (Molecular Research Center, Cincinatti, OH, USA) and stored at −20 °C before RNA isolation.

### 4.4. RNA Separation Methods

#### 4.4.1. Isolation of Cell-Free microRNAs from the CSF Samples

Thawed CSF samples were first centrifuged at 10,000 g for 1 min and 200 µL of cell-free CSF supernatants were spiked-in with 10 pmol mirVana cel-miR-39 mimic (Ambion, Austin, TX, USA, ID:MC10956). For cell-free miRNA extraction, the miRNeasy Serum/Plasma Advanced Kit (Qiagen, Hilden, Germany) was used according to the manufacturer’s recommendations.

#### 4.4.2. Total RNA Extraction from HCPEpiCs

Total RNA including intracellular miRNAs was isolated from HCPEpiC cells using the TRI Reagent (Molecular Research Center) according to the manufacturer’s recommendations. The purity and concentration of separated RNA samples were verified by a NanoDrop 2000 spectrophotometer (Thermo Scientific, Wilmington, DE, USA) and samples were stored at −80 °C before real-time quantitative polymerase chain reaction (RT-qPCR) analysis.

### 4.5. RT-qPCR Analysis of microRNAs and Messenger RNAs

#### 4.5.1. MicroRNA-Specific RT-qPCR Technique

The expression of extra- and intracellular miRNAs (miR-223-3p, miR-155-5p and miR-181b-5p) was quantified by Universal ProbeLibrary (UPL) probe-based stem-loop RT-qPCR assays [23,36]. This quantitative method included two steps: (i) miRNAs were transcribed into cDNA via miRNA-specific reverse transcription (RT) using TaqMan MicroRNA Reverse Transcription kit (Applied Biosystems, Vilnius, Lithuania) with specific stem-loop RT primers (500 nM, Integrated DNA Technologies, Leuven, Belgium) and (ii) miRNA quantification was performed by RT-qPCR using miRNA-specific designed forward primer (100 µM, Integrated DNA Technologies), universal reverse primer (100 µM, Integrated DNA Technologies) and FAM-labeled UPL probe #21 (10 µM, Roche Diagnostics) with recombinant Taq DNA polymerase (5 U/µL, Thermo Scientific, Vilnius, Lithuania) and dNTPs (2.5 mM, Thermo Fisher Scientific). Measurements were run in triplicates on a QuantStudio 12K Flex RT-qPCR instrument (Applied Biosystems by Thermo Fisher Scientific, Waltham, MA, USA). The reactions were incubated at 95 °C for 1 min, followed by 40 cycles of 95 °C for 15 s and 60 °C for 30 s [23,36,37,38]. The exogenous spike-in control cel-miR-39-3p was measured with the same method as above for the normalization of extracellular miRNAs. For intracellular miRNA analysis, the small-nucleolar RNU-43 was used for normalization. Oligonucleotides and qPCR assays were designed by the software developed by Czimmerer et al. [37], and sequences of primers that were used in this study are listed in Supplementary Table S1.

#### 4.5.2. RT-qPCR Analysis of Messenger RNAs

For the quantification of selected mRNAs (HMOX1, IL8, ICAM1, and VCAM1), cDNA synthesis was performed using the High Capacity cDNA Reverse Transcription Kit (Applied Biosystems, Vilnius, Lithuania) according to the manufacturer’s recommendations. The initial amount of RNA was 1000 ng per reaction in the case of HCPEpiCs. RT-qPCR was performed using a LightCycler 480 SYBR Green I Master mix (Roche Diagnostics) with gene-specific primers (10 µM, Integrated DNA Technologies). The reactions were run on a LightCycler 96 RT-qPCR instrument (Roche Diagnostics). The reactions were incubated at 95 °C for 10 min, followed by 40 cycles of 95 °C for 10 s and 60 °C for 1 min [36,38]. For normalization, we used the reference gene RPLP0 (36B4). Sequences of these oligonucleotides are listed in Supplementary Table S1.

### 4.6. Investigation of NF-κB Pathway Activation in HCPEpiCs Induced by Post-IVH CSF

#### 4.6.1. Detection of NF-κB p65 Subunit Nuclear Translocation

The NF-κB pathway activation (p65 nuclear translocation) in HCPEpiC cultures was visualized via p65 nuclear immunofluorescence staining based on the method drawn from our previous study [38] with some minor modifications. For this purpose, HCPEpiCs were cultured in BioLite 24-well plates (Thermo Scientific) on sterile glass microscope slides at a density of 5 × 10^4^ cells/slide. Cells were then treated with IVH-III, IVH-IV, or non-IVH control CSF samples (10 *v*/*v* %) for 1 h. After treatment, cells were washed twice with DPBS solution and fixed with ice-cold methanol-acetone (50 *v*/*v* %, Sigma-Aldrich, St. Louis, MO, USA) for 10 min. Non-specific antibody binding sites were blocked with FBS (Sigma-Aldrich) for 15 min. For primary labeling of the NF-κB p65 subunit, a polyclonal rabbit anti-human p65 antibody (100 μg/mL, sc-372, Santa Cruz Biotechnology, Dallas, TX, USA) was used for 1 h, followed by secondary staining with Alexa Fluor 488-conjugated goat-anti-rabbit IgG (5 μg/mL, A32731, Invitrogen, Carlsbad, CA, USA) for 1 h. Cell nuclei were labeled with 4′,6-diamidino-2-phenylindole (DAPI, Invitrogen) and samples were observed by Zeiss Axio Scope A1 fluorescent microscope (HBO 100 lamp) (Carl Zeiss Microimaging GmbH, Goettingen, Germany); DAPI: excitation at 365 nm and emission BP filter 445/50 nm; and fluorescein: excitation of BP filter at 470/40 nm and emission BP filter 525/50 nm. Images were analyzed with ZEN 2012 v.1.1.0.0. software (Carl Zeiss Microimaging GmbH). The ratio of nuclear and cytosol fluorescence intensity was calculated for NF-κB p65 staining. The specificity of immunostaining was checked by incubating the cells with the secondary antibody alone and only a limited background staining was seen.

#### 4.6.2. Analysis of the NF-κB Signaling-Dependent Inflammatory Response in HCPEpiCs Using a Specific Pathway Inhibitor

The NF-κB pathway inhibitor BAY 11-7082 (Selleck Chemicals, Houston, TX, USA) was used to investigate the role of NF-κB pathway signaling in stimulated HCPEpiC cells. For this purpose, HCPEpiC cells (2 × 10^5^ per well) were pretreated with the specific NF-κB pathway inhibitor BAY 11-7082 (5 µM) or DMSO (vehicle) for 4 h and cells were then exposed to IVH-III, IVH-IV, or control CSF samples (10 *v*/*v* %) for 24 h. Afterwards, cells were washed once with sterile DPBS solution, lysed in 1 mL TRI Reagent, and pro-inflammatory mRNAs were quantified with the same method mentioned above.

### 4.7. Measurement of Soluble Protein Markers by ELISA

To perform enzyme-linked immunosorbent assay (ELISA), the CSF samples were first centrifuged at 10,000× *g* for 1 min at room temperature. Soluble IL-8 (D8000C), TNF-α (DTA00D), VCAM-1 (DVC00), and ICAM-1 (DCD540) protein concentrations were measured by commercially available ELISA kits based on the manufacturer’s protocols (R&D Systems, Minneapolis, MN, USA).

### 4.8. Cell Viability Assay

To assess the cell viability after the CSF treatments, a Thiazolyl Blue Tetrazolium Bromide (MTT)-based cell viability assay (Sigma-Aldrich) was performed [10]. HCPEpiCs (10,000 per well) were subcultured into PLL-precoated 96-well plates (SPL Life Sciences, Naechon-Myeon, Korea) and then exposed to IVH-III, IVH-IV, or non-IVH CSF samples (10 *v*/*v* %) for 24 h. After treatment, supernatants were removed and 100 µL of 3-(4,5-Dimethyl-2-thiazolyl)-2,5-diphenyl-2H-tetrazolium bromide (MTT) solution in Hank’s Balanced Salt Solution (HBSS, Sigma-Aldrich) was added to each well. After incubation for 3 h (37 °C, 5% CO_2_, humidified atmosphere), the MTT solution was discarded and blue formazan crystals were solubilized in 100 µL dimethyl sulfoxide (DMSO, Sigma-Aldrich), and optical density was determined at 570 nm.

### 4.9. Intracellular ROS Measurement

Intracellular ROS production was measured by a CM-H_2_DCFDA-based General Oxidative Stress Indicator assay (Invitrogen). For this purpose, HCPEpiCs (10,000 per well) were sub-cultured into PLL-coated 96-well plates (SPL Life Sciences) and confluent cells were exposed to IVH-III, IVH-IV, or non-IVH control CSF samples (10 *v*/*v* %) for 1 h and 4 h. After treatment, cells were washed with HBSS solution (Sigma-Aldrich) two times and then loaded with CM-H_2_DCFDA (10 µmol/L) for 30 min at 37 °C in the dark. The fluorescence intensity was detected every 30 min for 3 h by applying 488-nm excitation and 533-nm emission wavelengths using a Beckman Coulter DTX 880 analyzer (Beckman Coulter, Pasadena, CA, USA).

### 4.10. Statistical Analyses

The Kolmogorov–Smirnov test was used for the evaluation of the normality of data. Results are expressed as mean ± SD or SEM and median with the interquartile range (IQR) as appropriate. To compare the data of the two groups, we applied the unpaired *t*-test or Mann–Whitney U test and chi-squared test. The comparison of multiple groups was performed using ANOVA with Bonferroni’s multiple comparisons test or Kruskal–Wallis test with Dunn’s multiple comparisons test as appropriate. Correlations between the total heme levels and the soluble pro-inflammatory biomarkers were determined using Spearman’s test. Statistical significance was defined when the *p* value was <0.05. Statistical analyses were performed using GraphPad Prism software (version 6.01, La Jolla, CA, USA).

## 5. Conclusions

In preterm IVH, the function of choroid plexus epithelial cells is substantially influenced by the accumulated pro-inflammatory and pro-oxidant mediators in the subarachnoid space, resulting in a massive inflammatory response in these cells. These events may contribute to the development of long-term complications of the disease that must be investigated in future follow-up clinical studies. Our current study suggests that pharmacological intervention against extracellular Hb oxidative derivates and heme may limit the neuroinflammation after IVH.

## Figures and Tables

**Table 1:** Baseline demographical and routine laboratory characteristics of IVH patients and clinical controls (*n* indicates the number of involved subjects). Data are expressed as mean ± SD or median with IQR. For statistical analyses, chi-square test and unpaired *t*-test or Mann–Whitney U test were used, as appropriate.

Patient’s Parameters	Non-IVH Patients (*n* = 14)	Grade-III IVH Patients (*n* = 21)	Grade-IV IVH Patients (*n* = 26)
Gestational age (weeks)	32.0 ± 6.8	29.1 ± 4.2	27.5 ± 1.9
Birth weight (g)	2046 ± 1281	1395 ± 800	1108 ± 376
Gender (female/male)	8/6	9/12	13/13
Whole blood RBC (T/L)	3.7 (3.4–4.6)	3.9 (3.4–4.3)	3.8 (3.1–4.4)
Whole blood Hb (g/L)	107 (99–130)	121 (109–144)	118 (100–133)
Whole blood WBC (G/L)	10.3 (8.6–12.3)	12.2 (10.8–15.3)	11.5 (9.3–12.9)
Whole blood platelet (G/L)	370 (288–509)	384 (296–481)	395 (282–507)
Serum CRP (mg/L)	0.5 (0.5–4.5)	1.2 (0.7–4.8)	1.7 (0.6–4.1)
Serum PCT (µg/L)	0.2 (0.1–0.2)	0.3 (0.2–0.5)	0.3 (0.2–0.6)

Abbreviations: CSF, cerebrospinal fluid; CRP, C-reactive protein; Hb, hemoglobin; IVH, intraventricular hemorrhage; PCT, procalcitonin; RBC, red blood cell; and WBC, white blood cell.

## Data Availability

The data presented in this study are available on request from the corresponding author.

## Supplementary Materials

The following are available online at https://www.mdpi.com/article/10.3390/ijms22168648/s1: Figure S1. Correlation analysis of CSF total heme level with other soluble parameters; and Table S1. Sequences of primers for the analysis of mature microRNAs and messenger RNAs. In the case of stem-loop reverse transcription primers, the miRNA specific sequences are highlighted in red. Universal ProbeLibrary probe #21 (5′-TGGCTCTG-3′) was used for the RT-qPCR measurements.
