# Supplementary material for: Preterm Intraventricular Hemorrhage-Induced Inflammatory Response in Human Choroid Plexus Epithelial Cells
Source: Int J Mol Sci. 2021 Aug 11;22(16):8648. doi: 10.3390/ijms22168648 (PMC8395401; doi:10.3390/ijms22168648)
Supplement: Supplementary file 1 [file ijms-22-08648-s001.zip › Suppl. Figure1_Fejes_IJMS_2021_R1.pdf]

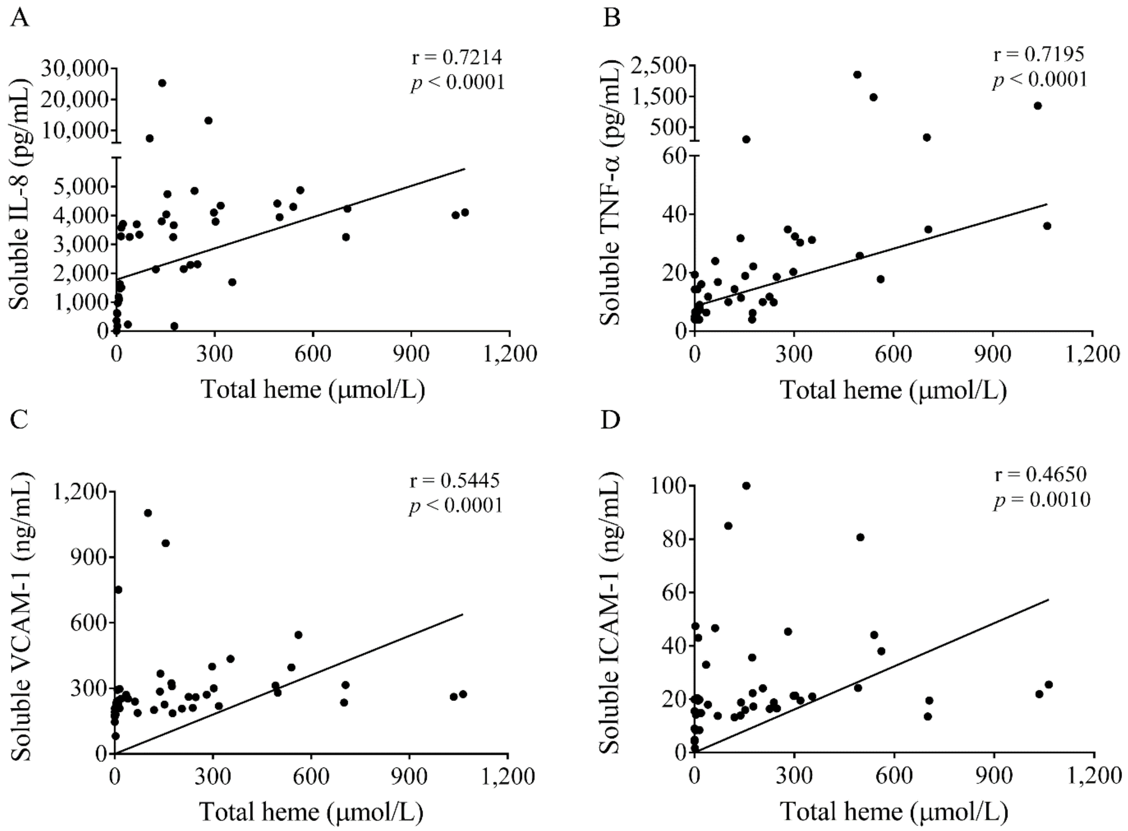

Figure S1. Correlation analysis of CSF total heme level with other soluble parameters. Correlations between total heme levels and other soluble parameters measured in IVH CSF samples (n = 47) were determined using Spearman's test. Strong correlations were found between total heme levels and soluble IL-8 (A), TNF-α (B), VCAM-1 (C), and ICAM-1 (D) concentrations in CSF samples. CSF: cerebrospinal fluid, ICAM-1: intercellular adhesion molecule 1, IL-8: interleukin-8, IVH: intraventricular hemorrhage, TNF-α: tumor necrosis factor alpha, VCAM-1: vascular cell adhesion molecule 1.
